# The therapeutic promise of ATP antagonism at P2X3 receptors in respiratory and urological disorders

**DOI:** 10.3389/fncel.2013.00267

**Published:** 2013-12-19

**Authors:** Anthony P. Ford, Bradley J. Undem

**Affiliations:** ^1^Afferent Pharmaceuticals Inc.San Mateo, CA, USA; ^2^Allergy and Clinical Immunology, Johns Hopkins University School of MedicineBaltimore, MD, USA

**Keywords:** P2X3, AF-219, visceral disorders, afferent sensitization, airways hyperreactivity, cough, urinary symptoms

## Abstract

A sensory role for ATP was proposed long before general acceptance of its extracellular role. ATP activates and sensitizes signal transmission at multiple sites along the sensory axis, across multiple synapses. P2X and P2Y receptors mediate ATP modulation of sensory pathways and participate in dysregulation, where ATP action directly on primary afferent neurons (PANs), linking receptive field to CNS, has received much attention. Many PANs, especially C-fibers, are activated by ATP, via P2X3-containing trimers. P2X3 knock-out mice and knock-down in rats led to reduced nocifensive activity and visceral reflexes, suggesting that antagonism may offer benefit in sensory disorders. Recently, drug-like P2X3 antagonists, active in a many inflammatory and visceral pain models, have emerged. Significantly, these compounds have no overt CNS action and are inactive versus acute nociception. Selectively targeting ATP sensitization of PANs may lead to therapies that block inappropriate chronic signals at their source, decreasing drivers of peripheral and central wind-up, yet leaving defensive nociceptive and brain functions unperturbed. This article reviews this evidence, focusing on how ATP sensitization of PANs in visceral “hollow” organs primes them to chronic discomfort, irritation and pain (symptoms) as well as exacerbated autonomic reflexes (signs), and how the use of isolated organ-nerve preparations has revealed this mechanism. Urinary and airways systems share many features: dependence on continuous afferent traffic to brainstem centers to coordinate efferent autonomic outflow; loss of descending inhibitory influence in functional and sensory disorders; dependence on ATP in mediating sensory responses to diverse mechanical and chemical stimuli; a mechanistically overlapping array of existing medicines for pathological conditions. These similarities may also play out in terms of future treatment of signs and symptoms, in the potential for benefit of P2X3 antagonists.

## Introduction

ATP is an abundant, multifaceted molecule: the chemical capital stoking metabolism in every cell; ubiquitous transmitter and autocrine signal; extracellular herald of movement, distension, distress, ischemia, damage and inflammation (Burnstock, 2012). Moreover, ATP can foment irritation, pain and discomfort, provoking maladapted autonomic reactions (Burnstock, 2012; Ford, 2012; North and Jarvis, 2013). The mechanisms by which ATP is liberally and specifically discharged by cells during this process are now being delineated, with evidence for participation of processes akin to synaptic vesicle release, hemichannel efflux, and extrusion through ligand gated channels, in addition to the spillage of copious ATP during cell distress and rupture (Burnstock et al., 2012). In a variety of pathological settings, these release mechanisms may operate more aggressively, or alternatively enzymatic disposition mechanisms (nucleotidases) become weakened, leading to elevations in background and stimulated ATP concentrations in extracellular milieu. Such settings include inflammatory diseases (arthritis), ischemia, cancer, airways pathology, and bladder disorders, with the associated implication that excess ATP *per se* contributes to heightened sensations that attend these disorders (Ford, 2012).

This commentary focuses on the P2X regulation of primary afferent neurons (PAN), which link sites in the peripheral receptive field to the first synapse of the sensory pathway in the spinal dorsal horn and dorsal brainstem, and in particular how they process signals from hollow organs. PANs have their cells bodies in the dorsal root and cranial ganglia, their peripheral fibers en route to the receptive fields can be short or extremely long, and they exist in several distinctive types, with differential morphological properties, speeds of conduction and molecular markers and receptors. The diversity of sensorineuron types confers a wide range of functions from low threshold (non-nociceptive) proprioceptive, mechanosensitive and thermosensitive detection (mostly the faster fibers), to high threshold (nociceptive) fibers sensitive to noxious mechanical and/or chemical stimuli: some which transmit signals rapidly (A*δ*), and many others slowly (C). What is clear is that primary afferents are the first conduit for all sensory information, and thus the primary site that may undergo modulation and plasticity in chronic disease and injury, leading to persistently altered sensation or dysaesthesia (Basbaum et al., 2009; Burgess and Williams, 2010). ATP, acting via P2X3-containing receptors, is clearly able to modulate, perhaps even drive, some of these plasticity changes, and such findings may have ramifications in identifying novel therapeutics for a range of sensory maladies (Ford, 2012; North and Jarvis, 2013).

**Figure 1:**
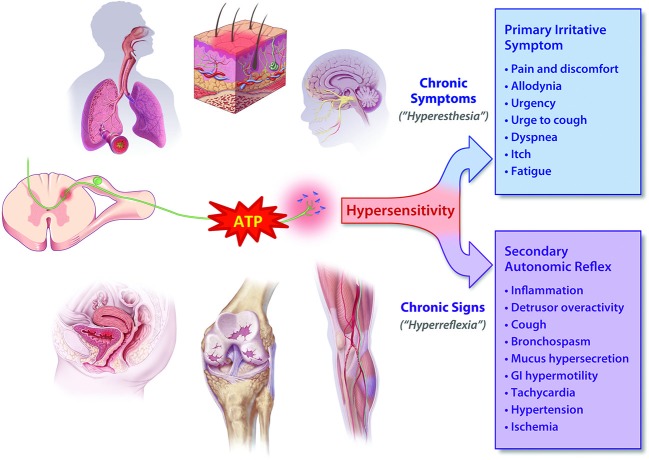
**ATP is released in heightened amounts in a variety of somatic and visceral tissue systems and may cause hyperexcitability (“sensitization”) of PANs**. Depending on the nature of the affected tissue, the elevated afferent discharge drives the increased perception of irritative symptoms (hyperesthesia) as well as lowering the threshold for activation of autonomic reflexes. These elevated reflexes (hyperreflexia) in turn give rise to many of the signs of chronic disorders, which can usually be easily observed or measured, if not perceived by the patient.

**Figure 2:**
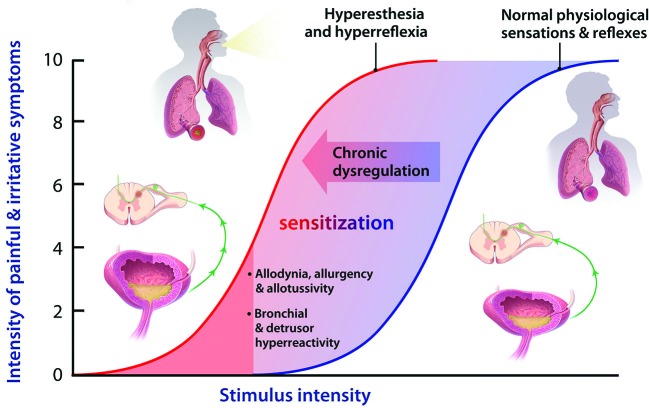
**Normal physiological sensory perception and reflexes are important defensive mechanisms, under conditions of acute stress or physical threat, when a high stimulus intensity (blue sigmoid) represents potential harm**. During chronic dysregulation, afferent functions experience sensitization, wherein normally low threshold or sub-threshold stimulus intensities, posing little or no threat, now induce unpleasant sensations and inappropriate autonomic responses. Many mechanisms have been proposed to contribute to such sensitization, but the key priming autacoid remains elusive, though it could turn out to be ATP in some visceral systems such as LUT and airways.

In considering the effects of P2X3 activation in visceral “tube and sac” (or “hollow-organ”) systems, our focus is placed on the lower urinary tract (LUT) and airways. These systems share many traits from a morphological, functional and therapeutic perspective (as discussed in more detail below), and are notable in that the most common pathologies in either system are associated with a range of primary symptoms and signs that include persistent and heightened, inappropriate irritative sensations and exaggerated autonomic reflexes, as depicted in Figure 1. To date, the primary sensory causes of both hyperesthesia and hyperreflexia in these systems have remained unclear, and have been therapeutically intractable and/or underexplored. Our suspicion is that this situation may be on the threshold of a significant advance, with the arrival of selective P2X3 receptor antagonists. Signaling via ATP-P2X3 seems to be not just another participant in the extracellular “soup” of transmitters, autacoids and inflammatory cytokines that contribute to pathologically suppressed sensory thresholds, but is a key common aggravator in the receptive field: promoting sensitization of PANs, priming them to many forms of chemical and physical stimuli that drive afferent excitatory traffic. Thus primed with lowered thresholds for activation, stimuli that would normally be perceived as innocuous are able to trigger inappropriately heightened and unpleasant sensations (hyperesthesia) and untoward responses (hyperreflexia), as illustrated in Figure 2. The components of this include: a convergent process whereby abundant irritative stimuli elicit increases in extracellular ATP concentrations, especially in disease; increased expression and cell-surface trafficking of the P2X3-containing receptors on PAN endings (Giniatullin et al., 2008; Gnanasekaran et al., 2011); activation of key downstream excitatory pathways, such as CASK and PKC isoforms (especially PKCε), by these elevated ATP levels leading to reduced thresholds for activation of PANs by many other sensitizing stimuli (Parada et al., 2005; Gnanasekaran et al., 2013; Prado et al., 2013; Volonté and Burnstock, 2013). In focusing on the current topic of P2X3 participation in the sensitization of *peripheral* terminals of PANs in common sensory pathologies, it may be noted that we are potentially ignoring another possible key locus of sensitization: the central PAN terminals in spinal and brainstem dorsal horn. It is acknowledged that P2X3 receptors may participate in modulating the strength of synaptic communication with second order neurons (Gu and MacDermott, 1997) and that CNS penetrant antagonists may reduce sensory wind-up quite distal from the receptive field; however, for the focus of the current review these aspects will not be discussed further.

## The sensitizing property of ATP

Five decades ago it was reported that fluid from lysed red blood cells applied to exposed blister bases on the human forearm evoked pain and discomfort (Keele and Armstrong, 1964). In subsequent investigations (Collier et al., 1966; Bleehen et al., 1976; Bleehen and Keele, 1977), the candidate chemicals responsible were successively eliminated revealing that of the many chemicals discharged it is ATP itself that causes much of the pain. A generation later, in a reductionist version of the forearm studies, a co-culture of rat trigeminal afferents with skin cells was described (Cook and McCleskey, 2002): an electrode recorded activity of one neuron while a proximal keratinocyte was lysed yielding excitations following spillage of sensitizing cellular contents. The responsible chemical present in the lysate: ATP, acting—as was deduced in the interim—by opening P2X3-containing cation channels. These findings are consistent and profound: ATP is a key sensitizing autacoid.

The effects of ATP on blister bases can be mimicked by intradermal injection (Coutts et al., 1981), and by iontophoretic application to UV sensitized skin (Hamilton et al., 2000) in healthy human subjects. Intradermal ATP was also shown to sensitize cutaneous C-fibers (Hilliges et al., 2002). Subsequently, ATP was reported to produce moderate to strong pain and tenderness after intramuscular (trapezius) infusion in volunteers (Mork et al., 2003), which has recently been extended with a report of pain, ache and fatigue after injection into themar muscle (thumb pad; Pollak et al., 2013). Preclinical correlates of similar effects of ATP in rodents are also well described, based on “pain-related” or “nocifensive” responses evoked. For example, ATP (or *αβ*-MeATP) injection into the paw of rat evokes nocifensive responses (Bland-Ward and Humphrey, 1997; Hamilton et al., 1999, 2001; Tsuda et al., 2000). Similarly, ATP or *αβ*-MeATP injection into sensitized temporomandibular joint (TMJ) attenuates pressure thresholds (Shinoda et al., 2005), and into dental pulp sensitizes trigeminal afferents (Cherkas et al., 2012).

These effects of ATP on somatosensory systems form part of a significant body of evidence that P2X3 receptors contribute to increased nocifensive behaviors in many models of musculoskeletal and neuropathic pain, as extensively reviewed (see Khakh and North, 2006; Burnstock, 2013). The impact of selective antagonists to inhibit behaviors in these models is impressive allowing justified speculation about the potential for benefit in human musculoskeletal and neuropathic pain conditions (Jarvis et al., 2002; Ford, 2012; North and Jarvis, 2013). However, although somatic pain conditions seem to capture more attention, it is in the viscera, where sensory symptoms are so poorly addressed, that a greater breadth of evidence has evolved from a wide range of investigations that place ATP and the P2X3 receptor mechanism at the heart of pathological sensitization and where therapeutic potential may be most appealing.

In visceral systems, irritative direct effects of ATP have also been described: two reports have shown that inhalation of ATP can activate sensory responses in the airways driving the perception of symptoms (coughing, wheezing, dyspnea and chest-tightness; Basoglu et al., 2005) and bronchoconstriction (reduced FEV1; Pellegrino et al., 1996; Basoglu et al., 2005), with asthmatic subjects being more sensitive than healthy control subjects; it should be noted that on a molar basis ATP was found to be more potent than AMP, indicating that the effects were due to the inhaled ATP and not its dephosphorylated metabolite (adenosine). It has also been reported from several studies that intravenous infusions of ATP in pre-terminal cancer patients produces a common adverse effect in a large proportion of patients: chest discomfort/pain, dyspnea and the urge to take a deep breath (Haskell et al., 1998; Beijer et al., 2007). Some of these effects had been predicted based on a small number of preclinical evaluations examining ATP activation of canine pulmonary vagal afferents (Hurt et al., 1994) as well as participation in induction of cough in guinea pigs in response to tussive agents (Kamei et al., 2005; Kamei and Takahashi, 2006).

Unlike the situation in airways and somatic systems, no clinical reports indicate that ATP has been studied after its direct intravesical infusion, and thus it remains to be determined whether activation of sensation within urinary bladder could be so elicited. In animals, it clearly does produce marked local effects on afferent function after instillation (as described later), and this has been widely studied as a model of bladder irritability (Pandita and Andersson, 2002; Yu and de Groat, 2008; Ford and Cockayne, 2011). One thing that is consistently notable in all of the studies looking at ATP application in clinical and preclinical settings is that the effects of ATP on sensation (or afferent traffic) are greater when there is underlying irritation (a blister, UV and chemical insult) or pathology (asthma, bladder pain syndrome (BPS)).

## Morphological and wiring similarities of urinary tract and airways

**Figure 3:**
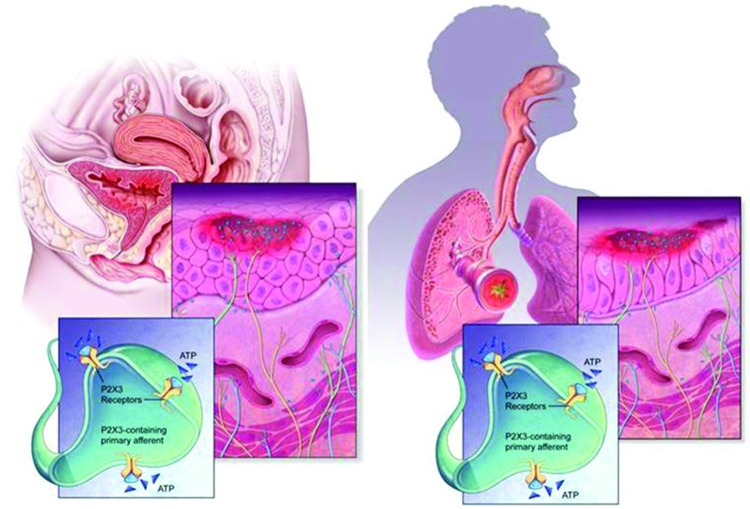
**Morphology and wiring of LUT and airways**. The urinary tract and airways walls show a similar overall morphology, despite quite distinct structural differentiation in the epithelial layer. In both systems, ATP (shown as blue triangles) is present in large extracellular concentrations, released by various cells including epithelia, fibroblasts and smooth muscles, and can activate C-fiber afferent and promote sensitization. Release of ATP is augmented in conditions of stress, injury, inflammation and infection.

The urinary bladder and airways walls show a significant organizational similarity, somewhat superficially reflected by the cartoon diagrams in Figure 3. In both tissues, a smooth muscle layer (more extensive in the case of the detrusor), under phasic excitatory control of parasympathetic efferent nerves, provides for compliance and tone during distension and constriction, and may become tonically activated in compliance disorders such as asthma and overactive bladder (OAB). This muscularis is anchored by a layer of cartilage in the airways (of decreasing presence with narrowing of the airway branches) and by serosal fat, fibrous connective tissue and peritoneum in the LUT. The smooth muscle itself is layered by a submucosa, or lamina propria, which harbors many connective tissues, vascular plexuses, inflammatory, intrinsic modulatory (myofibroblasts) and secretory cells. The most luminal layer has its margin as the basement membrane, and supports the epithelium, which in the airways is a pseudostratified columnar epithelium with many cells ciliated on the apical surface; and in urinary bladder is a transitional epithelium, comprising a basal cell, 3–4 transitional cells, and a highly specialized apical “umbrella” cell that is coated with unique proteins call uroplakins. Clearly the apical epithelial differentiation contrasts sharply between airways and bladder, reflecting the starkly different physiological needs regarding permeance: airways are obligatorily designed for chemical and fluid exchange, whereas the LUT uses its apical, uroplakin-decorated surface as a primary barrier to limit permeance from lumen to parenchyma (although this barrier function is often undermined by infections and in chronic diseases such as BPS; see Wang et al., 2005).

What appears to be intriguing in each tissue system is that the epithelium plays a key role not only in protecting the tissue from “the outside world”, providing resistance to pathogens and defense against chemicals, but also in sensing changes (pressure, movement, irritation) and signaling to adjacent afferent nerve endings. In this latter respect, it is well established that copious release of ATP accompanies such environmental changes, and this ATP communicates with the afferents via P2X3 receptor activation.

On a simplified level, the wiring control of these organ systems also exhibits similarities. In the LUT, sensory afferents of the pelvic, pudendal and splanchnic (hypogastric) nerves, with the unmyelinated C-fibers in significant numerical predominance over A*δ*, convey much of the ongoing information to the CNS, where primary coordination is modulated at the pontine level. Efferent excitatory function to support voiding is largely carried by pelvic nerve parasympathetic fibers, with sympathetic innervation of the bladder neck and urethra providing the autonomic support during the long periods of continence, coupled with suspension of parasympathetic drive and maybe some sympathetic activity maintaining detrusor muscle compliance during filling. A large proportion of C-fibers and some A*δ*-fibers from the LUT express P2X3 subunits and respond to ATP.

The upper and lower airways are densely innervated by sensory and autonomic fibers. Although a small percentage of fibers originate from dorsal root ganglia (DRG), most afferent fibers are carried by the vagus nerve, with cell bodies contained in the nodose (epibranchial placode derived) and jugular (neural crest derived) cranial ganglia (Undem et al., 2004). These embryological derivations lead to differentiation also in the properties of the numerically predominant populations of afferents, the C-fibers. For example, the proportion of fibers containing neuropeptides is high in DRG and jugular and is low in nodose. Similarly, there is differentiation in terms of P2X3 containing receptors, such that nodose fibers express P2X2 and P2X3 subunits, and likely carry responses by P2X2/3 heterotrimers, whereas neural crest derived afferents express P2X3 and little P2X2, and respond via the P2X3 homotrimer, at least in rodents (Undem et al., 2004; Kwong et al., 2008; Nassenstein et al., 2010).

How all these elements militate together and integrate their participation in organ function has been studied extensively over decades, using all sorts of *in vivo* and deconstructed systems; of these, one type of approach has offered greatest perspicacity: the isolated tissue-nerve preparation, as further discussed below.

## Tube and sac function and dysfunction: lower urinary tract

The functions of the LUT, dominated by the passive, low pressure storage of volumes of urine, irregularly interrupted by brief episodes of coordinated micturition, operate by what seems a simple switch operated process: long periods of detrusor compliance and expansion coupled with sympathetically maintained bladder neck closure and urethral coaptation are conveniently—and preferably consciously—awoken by a parasympathetically driven coordinated detrusor muscle contraction and urethral/sphincteric relaxation with parallel suspension of sympathetic and somatic motor drive. Outflow of these efferent autonomic signals are regulated by pontine storage/micturition centers (Barrington’s nuclei), in turn under descending cortical control (Fowler et al., 2008; de Groat and Wickens, 2013). This latter factor is somewhat unique for a visceral organ system.

**Figure 4:**
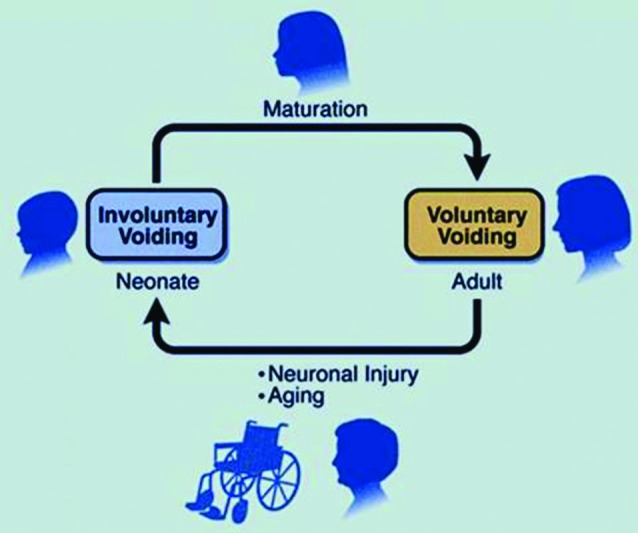
**The reflex bladder**. In the neonate, C-fibers carry bladder filling signals to activate spinal segmental reflexes that regulate involuntary excitatory responses. Overlaying and—in a healthy person—overriding this reflex bladder is a voluntary control system, that is laid down during the early post-natal years. Here, A*δ* fibers play a dominant role, impacting with second order neurons that send signals up to the brain. In neurogenic-bladder patients (typified following spinal injury), a rapid deterioration of this descending inhibitory control occurs, ”unmasking” the C-fiber reflex beneath. The more gradual emergence of this reflex, due to idiopathic loss of descending C-fiber inhibition, may account for the development of many LUT symptoms (as conceived by WC de Groat).

The afferent limb of urine storage and elimination displays a dualism, both structurally and functionally. Two operationally and neurally distinct paths appear to sense the condition of the LUT, and relay this information to spinal and supraspinal circuits, via the periaqueductal gray (PAG) to pontine control centers (de Groat and Wickens, 2013). One of these uses in particular thinly myelinated A*δ* fibers to detect volume expansion at high thresholds (via stretch and chemical receptors) in the bladder wall and conveys this information to the pontine centers and beyond, to inform conscious and graded perception (sense of fullness, developing urge to void, extreme urge to void) and prepare consciously integrated autonomic reflex coordination. The second system, perhaps a more primitive one, engages unmyelinated C-fibers and detects filling at a broader range of volume thresholds, as well as signals of local distress (infection, inflammation), and can elicit local (spinal segmental) initiation of efferent autonomic responses that lead to increased detrusor activity. de Groat (1997) has described this C-fiber system as the “reflex bladder” circuit, in that it operates without conscious control. In a healthy adult these latter afferent signals are considered to be under considerable descending inhibition that is established purposely during early development of conscious voiding control (de Groat, 1997). Accordingly, a healthy adult relies mostly on the first (A*δ*) pathway to ascertain bladder status and desire to void, with C-fiber signals failing to escape tonic suppression except when local pathological conditions arise (such as in infection). The “reflex bladder” (Figure 4) may represent the state dominant in the infant, before descending control emerges, and that can abruptly return during urinary tract infections (where the discharge to segmental circuits is too great to suppress), as well as after the erosion of the descending inhibitory influences that can occur abruptly (spinal injury, stroke) or gradually, as in neurodegenerative disease, or even aging (de Groat, 1997).

It has been suggested that the common urological conditions associated with irritative LUT symptoms (LUTS): urgency, frequency, nocturia, incontinence, discomfort and even pain are mediated by the unsilencing of these C-fiber pathways and emergence of the reflex bladder. Thus, an optimal approach would be to target selectively these afferents for suppression, to provide relief for countless numbers of LUTS sufferers with conditions such as OAB, benign prostatic hyperplasia (BPH), BPS and chronic pelvic pain syndrome (CPPS): all representing forms of pelvic hypersensitivity, typified by inappropriate urgent sensations at modest filling that trigger unwelcome efferent responses (hyperreflexia). Ideally, an understanding of what substances (cytokines, trophic factors) cause the unsilencing of the reflex bladder would be additionally helpful.

## Tube and sac function and dysfunction: the airways

As was described for the LUT, the airways depend upon the coordination of afferent inputs to central (in this case medullary) nuclei to regulate the critical and extensively rhythmic efferent autonomic influences upon upper and lower respiratory tissues. Given the functional complexity of airways and the second by second need to modulate and tune functions, exquisite surveillance of the tissues (and the blood gases) and timely modulation of efferent responses are all the more critical to homeostatic wellbeing, and thus the complexity of afferent inputs is amplified.

What is clear is that the airways need to respond appropriately to subjectively perceived irritations (particulate or chemical) as well as the perception of “air hunger”. Likewise, they must respond to afferent signals with well-regulated autonomic reflexes that may variously govern airways caliber and conductance (bronchoconstriction and dilation), fluid and mucus secretion and ciliary clearance. As described for the LUT, very common airways pathologies arise that are associated with poorly coordinated afferent-efferent communication, inappropriate sensations (hypertussive perceptions and dyspnea) and markedly reduced sensory thresholds that lead to maladaptive reflexes (airways hyperreactivity and hypersecretion). Variously, these signs and symptoms are common in chronic cough, asthma, COPD and interstitial disease, with hypersensitivity and hyperreflexia being key manifestations of widespread diseases. Although these diseases may have etiological bases in immune dysregulation and chemical exposure, it remains clear that current therapeutics offer merely patchy resolution of some but not all signs and symptoms. Though the underlying causes may largely drive changes through inflammatory pathways and factors, it remains clear that afferent targets in the respiratory system could offer important opportunities for therapeutic intervention.

## Tissue-nerve preparations

An approach that has been employed to impressive effect in the study of both LUT and airways to elucidate the origination and composition of afferent nerve responses to mechanical and chemical stimulation signals is the intact “tissue-nerve” preparation from rodents. The bladder-nerve preparations (as originally defined by Morrison and colleagues: Namasivayam et al., 1998; Morrison, 1999) and the airways-nerve preparations (in various forms, as described by Undem and Colleagues: Myers et al., 1991; Riccio et al., 1996; McAlexander et al., 1999; and by Fox et al., 1993, 1995) have provided interesting insights to the types of sensory fibers activated by distinct stimuli and what chemical messengers, receptors and channels mediate them. The advantages of these in vitro preparations are nicely described in the respective reports: they allow for study of chemical application and sensitization, and controlled mechanical force and volume displacement at the level of the airway or urinary tract “receptive” nerve endings, but where such variables can be isolated from the impact of descending nervous modulation and hemodynamic or inflammatory factors. They also allow for more precise direction of the nature and location of stimuli, and allow control over concentrations of exogenous irritants and modulators. The results from these studies have been very revealing. Most particularly, these preparations allow for study of maneuvers and chemical stimuli that directly effect changes in afferent discharge as well as those that may act directly but function to modulate or sensitize afferents to other stimuli (e.g., sensitizing autacoids; Myers et al., 1991). In the context of the ATP-P2X3 mechanism, the findings have been insightful.

In the LUT preparations (mostly bladder/urethra—pelvic nerve, but also ureter—pelvic/hypogastric nerve), Namasivayam et al. (1999) were the first to follow the bold proposal (from Ferguson et al., 1997) that the distension induced release of copious ATP from urothelial cells in bladder reflected a role for local release of the nucleotide in signaling to primary afferents on the status of bladder filling. They showed quite clearly that the multi-unit recordings from pelvic nerve during distension were largely (> 50%) dependent on ATP receptors, by showing that treatment of the distending bladder with the nonselective P2 antagonist suramin reduced firing by a half, and that *αβ*-MeATP desensitization led to 65–75% reduction of nerve firing. Subsequently, it was shown that bladders from P2X3 gene knockout and P2X2–P2X3 double knockout mice would distend with much larger volumes of fluid before significant response was observed in pelvic nerve recordings (Vlaskovska et al., 2001; Cockayne et al., 2005). The role of ATP mediating pelvic nerve firing during filling has been explored by many groups, and it is clear now that certain subpopulations of afferent nerves may mediate this specific response, that the effects are sensitive to blockade with P2X3 selective antagonists (Rong et al., 2002; Zagorodnyuk et al., 2007, 2009; Sun et al., 2012) and in particular that the contribution of this receptor function is upregulated in disease models (Yu and de Groat, 2008, 2010; Sun et al., 2012); this occurs possibly due to increased receptor expression and coupling, and/or an elevation in the amount of ATP that is discharged by distension. Most recently, the beneficial impact of botulinus toxin (onabotulinumA) on sensory symptoms in OAB have been modeled in a mouse bladder-nerve preparation illustrating that intravesical exposure of the toxin reduces both ATP release during distension and afferent nerve discharge (pelvic and hypogastric; Collins et al., 2013). Given this seemingly unique role of the ATP-P2X3 mechanism in the LUT, it is not surprising that selective antagonism of this receptor population represents a breakthrough opportunity for management of conditions where sensory symptoms (urgency, frequency, nocturia, pain) and attenuated thresholds for initiation of reflex detrusor excitability are common.

In the airways, focus on ATP as a sensitizing autacoid or transmitter has been limited, indeed more focus has been placed on its potential roles in inflammation, ciliary motility and mucus clearance (Idzko et al., 2007; Cicko et al., 2010; Koeppen et al., 2011). However, our recent efforts to examine the contribution of ATP-P2X3 signals to afferent excitation generated evidence for significant convergence of stimuli onto this target. Using an isolated perfused lung-vagus nerve preparation (Weigand et al., 2012), it was seen that, consistent with previous reports, methacholine and histamine were both able to produce bronchoconstriction and action potential discharge in nodose derived vagal fibers. ATP was also able to activate nodose fibers, but without eliciting changes to perfusion resistance, indicating a lack of direct effect on smooth muscle tone. The effects of ATP were inhibited by two chemically distinct P2X3-P2X2/3 antagonists, TNP-ATP and AF-353, which also were both able to inhibit the excitatory effects of methacholine and histamine on nodose neurons, though leaving the constrictor responses unaffected. The implication, that ATP is the mediator of the indirect neural responses to the two spasmogens, was also in evidence by the loss of neural responses if apyrase was present. Thus, ATP is a necessary intermediate for bronchoconstriction-induced nodose C-fiber excitation; the fact that ATP is unable to elicit action potentials of jugular-derived vagal afferents also explains why jugular C-fibers are not activated by histamine (Undem et al., 2004; Kwong et al., 2008). These dramatic findings put ATP in the spotlight as a key sensitizer of airways afferents under a variety of physiological circumstances, and raise the possibility that specific antagonism of P2X3-containing receptors may have a potential in several respiratory conditions where afferent activation and hyperreflexia drives both bronchial hyperreactivity and abundant sensory symptoms that are so poorly managed.

It is of significant note that the use of the LUT and airways “organ-nerve preparations” has allowed for the identification in each system of a major and shared pathway, that so crucially coordinates integrated signaling, and that might have been more difficult to reveal so clearly in the intact organism or by using a more reductionist approach. These isolated preparations allow for experimenter designed degrees of de-construction and signal isolation that can be examined and tested, then be re-constructed to understand their place in physiology and pathobiology.

## Current and future management of signs and symptoms in lower urinary tract and airways

Given the morphological and neurophysiological similarities between these organ systems, their dependencies on movement and compliance coupled with finely tuned parasympathetic coordination, it is perhaps not surprising that the current treatment options for millions of patients with signs and symptoms of disorder in either system share so many mechanistic features. Ironically, when one thinks about the pathophysiological underpinnings that have motivated decades of pharmaceutical discovery in these systems in the quest of novel, transformative therapies, the approaches could hardly be more different: in the airways, the immune system has received preponderant focus, with an abundance of enzyme, chemokine and cytokine targets and various immune and inflammatory cell types pursued as the cause du jour; in the LUT, it is the efferent neuroeffector influences and the apparently overexcitable smooth muscles that have historically been the focus. In neither case has decades of effort revealed a genuinely robust return in terms of transformational therapeutic attenuation of unmet need. Similarly, in both systems, the attention paid to the afferent circuits has been relatively insubstantial, especially given that the presenting disease burden represents such profoundly disturbing sensory experiences for so many of the patients: labored breathing, wheeziness, chest tightness, air hunger, urge to cough and cough itself; urinary frequency, persistent urgency, discomfort and pelvic pain, disturbed sleep (nocturia) and continence failure.

In the face of this significant sensory plight, the pulmonary and urological patients both have access to two mechanistic classes in common that aim to reduce the parasympathetic excitation of smooth muscles and improve compliance: antimuscarinics (for example: ipratropium, tiotropium and aclidinium for airways symptoms; oxybutynin, tolterodine and solifenacin for LUT symptoms) and *β* agonists (for example: albuterol, salmeterol and formoterol for airways symptoms; the newly developed mirabegron for LUT symptoms). The measure of clinical effectiveness of such agents within these systems is difficult to compare; however, the accessibility of the airways for inhalation therapy allows local delivery of drug concentrations imparting more meaningful clinical response than seems to have been achieved so far in LUT, where intravesical delivery is much less convenient or well-tolerated. Currently, systemic adverse events (AEs) greatly impair dosing to therapeutic effectiveness and frequently discourage LUTS patients from persisting with treatment. Whether this latter situation is improved with the new *β*3 adrenoceptor agonist will be followed closely.

Beyond these classes, airways patients also derive significant benefit from the local delivery of corticosteroids which go some way to reduce the inflammation and severity of some of the common symptoms (and indeed may even blunt afferent discharge). Such an option has been examined in the LUT, but here local approaches are not so feasible or convenient, and safety concerns with chronic systemic exposure would preclude routine steroid usage even if some benefit might be afforded (as has been reported for severe ulcerative forms of interstitial cystitis). In both systems, direct pharmacological targeting of sensory targets has been of limited value so far, except in some exceptional situations such as using inhalation delivery of local anesthetics (e.g., lidocaine) for intractable dyspnea in cardiac patients or their intravesical instillation for pain in BPS.

Thus, the current therapeutic options for patients whose lives are seriously impacted by abundant sensory symptoms from both organ systems do not directly target the C-fibers and their triggered reflexes; rather they focus on the efferent limb on the reflex: block the parasympathetic drive to smooth muscle excitability, or increase detrusor compliance by activating *β*-adrenoceptors receptors that are present (though in neither case likely well innervated). Overall, these approaches offer benefit but are potentially undermined by on-target AEs (especially, in the case of antimuscarinics, dry mouth and CNS effects). Clearly, there is a great need for something directed at quelling the sensory pandemonium.

## P2X3 antagonists—progression to clinic

So far, despite the efforts of a several pharmaceutical organizations (see Gum et al., 2012), only one medicinal candidate P2X3 antagonist has progressed into human studies (Ford, 2012). The aryloxy-pyrimidinediamine, AF-219 (Ford et al., 2013; Smith et al., 2013) is an orally active small molecule (Mol Wt. ∼350 Daltons) antagonist at human P2X3-containing receptors. The inhibitory potency (IC_50_) of AF-219 has been reported as ∼30 nM versus recombinant hP2X3 homotrimers and 100–250 nM at hP2X2/3 heterotrimeric receptors, potencies very similar to those reported for recombinant rat receptors, and it displays no inhibitory impact on any non-P2X3 subunit containing receptors (IC_50_ values ≫ 10,000 nM at recombinant homotrimeric hP2X1, hP2X2, hP2X4, rP2X5 and hP2X7 channels). Reports from other related chemical members of this P2X3 selective pyrimidinediamine class have shown that the mechanism of inhibition is non-competitive (allosteric) and have been mixed regarding species-independency of P2X3 receptor potency estimates: AF-353 (Gever et al., 2010) shows remarkable potency congruency between human and rat recombinant P2X3 homotrimers (IC_50_ values of 8.7 and 8.9 nM, respectively) whereas the more potent analog AF-792 (also referred to as RO-51; developed initially as a potential prodrug for AF-353) was shown to be less potent at human versus rat P2X3 receptors in one report (Serrano et al., 2012) and yet species-independent in another (Jahangir et al., 2009). It is important to note that some selectivity for P2X3 versus P2X2/3 channels has been a common claim across several chemical classes of inhibitors (see Gum et al., 2012: e.g., AF-219 analogs, nucleotides such as TNP-ATP, benzenetricarboxylic acids such as A-317491), although in most studies values reported are not affinity determinations but IC_50_ estimates. Under such circumstances true selectivity cannot be categorically inferred, especially for the competitive antagonists (such as TNP-ATP and A-317491) as the IC_50_ is a parameter that will change with agonist concentration used and depends on agonist potency at the different trimers.

To date, AF-219 has completed four Phase 1 (safety, tolerability and pharmacokinetic studies in normal healthy volunteers) and one Phase 2 (patient) studies, with 3 additional Phase 2 studies in progress.^1^ The three ongoing study are in osteoarthritic joint pain, BPS/interstitial cystitis and in asthmatic patients with results to be reported in mid-2014. The completed patient study was undertaken in patients with chronic, treatment-refractory cough, and was disclosed at the European Respiratory Society congress in September 2013 (Abdulqawi et al., 2013). In this 24 patient two (14-day) period, placebo controlled crossover study, AF-219 markedly and significantly reduced objective cough frequency: daytime cough rate –84% (95% CI –94 to –60; *p* < 0.001; per protocol analysis), and comparably reduced patients subjective cough related symptoms.

This was a relatively small pilot study, with a single, high daily dose level compared with placebo in patients with considerable cough burden. Nevertheless, the unprecedented magnitude of efficacy observed and its objective nature, coupled with the apparent absence of benefit on placebo, would appear to offer strong initial validation of the P2X3 target in a patient group with a significant “hollow-organ” sensory disorder, in keeping with the potential identified from preclinical in vitro and organ-nerve preparations. These findings also augur well for other signs and symptoms of airways disease that are impacted by afferent hyperexcitability, as well as important and bothersome conditions emanating from other tube and sac systems, including LUT. We await their outcomes with anticipation and excitement.

## Conflict of interest statement

Anthony Ford is an employee of Afferent Pharmaceuticals; Brad Undem is a member of the SAB of Afferent Pharmaceuticals.
